# Economic costs of veterinary drug and antibiotic use in commercial dairy cattle herds in Central European countries

**DOI:** 10.3389/fvets.2025.1714377

**Published:** 2026-01-07

**Authors:** László Ózsvári, Attila Dobos, Marietta Máté

**Affiliations:** 1 Department of Veterinary Forensics and Economics, University of Veterinary Medicine Budapest, Budapest, Hungary; 2 National Laboratory of Infectious Animal Diseases, Antimicrobial Resistance, Veterinary Public Health and Food Chain Safety, University of Veterinary Medicine Budapest, Budapest, Hungary; 3 Private Practitioner, Vác, Hungary

**Keywords:** antibiotics, antimicrobials, Central-Europe, classification of antibiotics, dairy cattle, indication of antibiotics, veterinary drug cost

## Abstract

Antimicrobial use in livestock production is a critical issue in terms of both animal health and public health, particularly due to its role in the emergence of antimicrobial resistance (AMR). In this study, the veterinary drug costs, with a particular focus on antibiotics, were surveyed in 20 commercial dairy cattle herds comprising a total of 18,653 cows in five Central-European countries (Czechia, Hungary, Serbia, Slovakia, and Slovenia) in 2019. The distribution of veterinary drug costs by product groups and indication, as well as the antibiotic usage by indication and class of active substance, were assessed. In 2019, the average veterinary drug cost per cow was €63.3, ranging from €29.0 in Czechia to €99.0 in Slovenia. Regarding the product groups the antibiotics were responsible for the largest cost share (40.8%; €25.8), followed by hormones (19.1%), surgical and medical products (13.7%), vaccines (13.1%), vitamins and supplements (8.7%), and antiparasitics (4.5%). By indication the highest proportions of total veterinary drug cost were related to mastitis (32.2%), reproductive disorders (22.9%), lameness (11.3%), and respiratory diseases (10.0%). Furthermore, 60.0% of the total antibiotic costs were used for mastitis treatments, followed by lameness (23.5%), respiratory and digestive diseases (11.5%), and reproductive failures (5.0%). Regarding antibiotic classes, cephalosporins alone represented 43.7% of the total antibiotic costs, followed by various combinations (e.g., penicillins and aminoglycosides, amoxicillin with clavulanic acid) at 21.2%, and penicillins alone at 9.6%. Fluoroquinolones, tetracyclines, macrolides were used to a lesser extent. In udder treatments, cefquinome, cefoperazone and cefapirin were the most widely applied antibiotics. Ceftiofur and cefquinome were frequently used for lameness cases, while tilmicosin, tulathromycin, and tildipirosin were common for respiratory and digestive disorders. Reproductive failures were predominantly treated with cefapirin and chlortetracycline. Our results show that Central European dairy cattle farms allocated the largest share of their veterinary drug expenditures to antibiotics, mainly due to the extensive use of intramammary infusions containing cephalosporins, penicillins, and aminoglycosides in mastitis treatments. However, considerable differences in veterinary drug and antibiotic use were observed between herds.

## 1 Introduction

Assessing and improving the health status of dairy cattle, as well as identifying the diseases that cause the greatest losses, are crucial for optimizing herd productivity. Herd health strongly influences production performance. Reducing costs, especially veterinary expenses, while improving product quality can enhance both profitability and productivity (1, 2). From a farm management perspective, factors such as herd size and location, along with preferences of producers and veterinarians regarding treatment options for specific diagnoses, play an important role. Moreover, the co-occurrence of diseases can interact in ways that further affect farm profitability. The high costs associated with disease treatment can substantially influence both herd performance and market outcomes (3).

The main diseases affecting dairy herd profitability include mastitis, reproductive disorders, lameness, and respiratory and digestive diseases (1, 2). Worldwide, mastitis is one of the most important diseases of dairy cattle, leading to substantial economic losses and adversely affecting milk quality. The widespread use of antibiotics in treating mastitis raises concerns about antibiotic resistance, antimicrobial residues in milk, and animal welfare, all of which are increasing concern to society (4). Globally, the annual economic burden of mastitis was estimated at US$ 19.7–30 billion in the dairy sector (5). In the United States, the cost of a single case of clinical mastitis in the first 30 days of lactation was reported to average around US$ 444 (6). In European dairy herds, the median total cost of an intramammary infection was estimated at around €230 per case (7). Experiences from Nordic countries further demonstrate that low antibiotic use can be combined with high milk yield and good milk quality, providing an example that effective herd management can reduce drug use without compromising productivity (8). According to Rasmussen et al. (9), the total annual global losses due to dairy cattle diseases were estimated at US$ 65 billion. Clinical mastitis (US$ 13 billion) and subclinical mastitis (US$ 9 billion) were identified as the costliest conditions, together accounting for approximately one-third of all global dairy disease-related losses.

Other common health problems, such as lameness, reproductive disorders and calf diseases, also have a considerable economic impact and drive veterinary drug use. Lameness reduces fertility and milk yield and increases the risk of premature culling (10, 11). The average cost per case of sole ulcer, digital dermatitis and foot rot was estimated at US$ 216.07, 132.96, and 120.70, respectively (11). Global economic assessments indicate that lameness contributes approximately US$ 6 billion annually to dairy-sector losses (9). Reproductive disorders such as endometritis reduce milk yield and prolong calving intervals, thereby contributing to higher veterinary drug expenditures (12–14). Global economic assessments indicate that metritis is among the costliest reproductive diseases in dairy cattle, contributing US$ 5 billion annually through reduced production and impaired fertility (9).

Infectious diarrhea in calves remains one of the most significant health challenges, accounting for approximately half of all calf mortalities in dairy herds (15). The estimated total cost per case varies between US$ 76 and US$ 533 depending on severity and farm system (16). Respiratory infections are another major health problem in calves and have a substantial economic impact. The frequency and severity of these infections have increased globally, influenced by factors such as the general health status, immune function, housing conditions, climate, quality of medication, and the spread of infectious agents (17–19). Moreover, diarrhea and pneumonia in dairy calves were estimated to cause economic losses of approximately US$ 242,000 in a single studied population due to mortality and treatment expenses (20).

Within the European Union, the European Medicines Agency (EMA) has developed the Antimicrobial Advice *Ad Hoc* Expert Group (AMEG) categorization system, which classifies antibiotics into four categories (A-D) according to their importance in human medicine and the associated risk of antimicrobial resistance (AMR). This framework emphasizes the need to restrict the use of critically important antibiotics and to promote prudent and responsible prescribing practices in food-producing animals (21). In line with these recommendations, a significant reduction in antibiotic use can be achieved on cattle farms, leading to improved disease resistance (22). In addition, the EMA's European Surveillance of Veterinary Antimicrobial Consumption (ESVAC) report provides comprehensive data on the sales and use of antimicrobials across EU member states, highlighting monitoring and reporting obligations (23). Recent analyses have also shown that European regulations increasingly emphasize responsible and restricted prescribing of antimicrobials in livestock production (24).

Investigating antibiotic use at herd level is of considerable importance. In Denmark, antimicrobial use in dairy herds is closely related to herd structure and calf introduction patterns, highlighting the role of management factors in shaping herd-level antibiotic use (25). In Sweden, analyses based on national veterinary treatment registers have shown generally low and selective antimicrobial use in dairy herds, with clear differences between organic and conventional systems and a strong emphasis on individual treatments and preventive measures (26). A survey of Slovak dairy herds described the prevalence of mastitis pathogens and their antimicrobial susceptibility, providing important evidence for rational drug use at the herd level (27). Analyses from Serbian dairy farms reported prevailing patterns of antibiotic use in mastitis therapy, underlining the need for diagnostics and more selective prescribing practices (28). In Slovenia, the predominance of smaller, family-based herds influences treatment practices and contributes to differences in antimicrobial use compared with other Central European countries (29). Although strategies to reduce antibiotic use are a priority, little progress has been made in reducing usage rates in livestock despite political initiatives and public pressure (30). Antibiotic use on farms can be reduced through timely clinical examinations, evaluation of animal welfare parameters, and the application of laboratory tests, which can help identify animals that are more susceptible to disease development (22).

The overarching goal of dairy farms is to maintain long-term profitability while adapting to market demands (31). Practical guidelines in dairy farming increasingly emphasize targeted antibiotic use, such as treating only infected cows based on herd-level data and diagnostic testing, rather than applying routine blanket treatments (32). In addition, international recommendations highlight the importance of disease prevention, the use of diagnostic tools, and regular herd health monitoring as key components of responsible antimicrobial stewardship (33). Hungarian dairy farms have also reported a growing adoption of non-antibiotic strategies, such as probiotics, as part of herd health programs aimed at reducing reliance on antimicrobials (34). In Czech dairy herds, culture-guided and on-farm diagnostics have been increasingly applied to support targeted mastitis treatment, reflecting practical antimicrobial stewardship (35).

The aims of our study were (1) to calculate the cost of veterinary medicinal products, including antibiotics, per cow on the basis of total drug expenditures in commercial dairy herds in Central European countries, and (2) to determine the distribution of veterinary drug costs and antibiotic use by product group and indication.

## 2 Materials and methods

### 2.1 Study design and data collection

A structured questionnaire was developed to collect information on veterinary drug use and costs, including antibiotics, in commercial dairy farms. The questionnaire was designed in collaboration with veterinary practitioners (*n* = 3) and university-based veterinary researchers (*n* = 3). A pilot version was tested with additional veterinary practitioners (*n* = 3), whose feedback was used to align the questionnaire with practical farm-level considerations and to improve its clarity and ease of completion before the survey was completed by potential respondents.

The participating farms were selected through convenience sampling. The inclusion criteria for commercial Holstein-Friesian dairy farms in the study were as follows: (1) a minimum herd size of 100 Holstein-Friesian cows to ensure sufficient treatment and drug-use data for reliable analysis (36, 37); (2) continuous participation in performance testing including milk recording; (3) use of computerized on-farm records including detailed records of veterinary treatments, drug use and associated costs, and (4) willingness to provide information and data to the authors. Farms that did not meet these criteria, or that had incomplete or inconsistent veterinary records, were excluded from the study.

This study employed a mixed-methods design that combined quantitative and qualitative approaches to data collection and analysis. The questionnaire consisted of structured, parameter-based items requiring respondents to provide numerical or factual information rather than selecting from predefined options. The first section collected general farm characteristics, including herd size (total and milking cows), number and type of barns, housing systems (e.g., freestall or deep bedding), milking technology (type, brand, capacity), and disease management practices (e.g., separation of sick animals, disease-free status). The second section focused on technological and production indicators, such as annual milk production and marketed milk volume, milk yield per lactation and per year, milk fat and protein content, average somatic cell count (SCC), lactation length and calving interval, and annual culling rate. The third section addressed veterinary drug costs, gathering information on the types and amounts of veterinary medicinal products used, their unit prices, and their distribution by product group and indication (e.g., udder diseases, lameness, respiratory diseases). This structure ensured the collection of consistent and comparable data across farms and formed the basis for subsequent cost analysis per cow.

The survey was conducted between February and September 2020 through structured personal interviews based on the questionnaire. Prior to the survey, all participating veterinarians provided written consent for the research. Participation was voluntary, and respondents remained anonymous. The obtained data were processed using Microsoft Excel™ 2016 (Microsoft Corporation, Redmond, WA, USA).

### 2.2 Data analysis

After data collection, the veterinary drugs purchased by each farm were categorized by product group and indication. Based on the quantity purchased and the unit price of each product per herd per year, the total expenditure for each drug type was calculated and subsequently aggregated to determine the overall drug costs across all surveyed farms. Considering the total number of cows, the annual veterinary drug cost per cow was also calculated for each country, enabling cross-country comparisons. Price data from Hungarian, Czech, and Serbian herds were provided in Hungarian Forints (HUF), Czech Koruna (CZK), and Serbian Dinars (RSD), and were converted into Euros (EUR) using the 2019 average exchange rates of the National Bank of Hungary (1 CZK = 12.67 HUF, 1 RSD = 2.76 HUF, 325.35 HUF = 1 EUR) (38). Cost data from Slovak and Slovenian herds were reported in Euros, therefore, no conversion was required.

Veterinary drugs were categorized into six product groups based on pharmacotherapeutic application: (1) antibiotics; (2) hormones; (3) vaccines; (4) surgical and medical preparations; (5) vitamins; and (6) antiparasitics (1, 2, 36, 39). The surgical and medical preparations group included the anti-inflammatories, analgesics, diagnostic materials and all other surgical and medical preparations that could not be classified into the remaining groups. Minerals, trace elements and amino-acid supplements were included in the vitamins group.

Veterinary drugs were also classified according to their indication (the specific disease or condition they were used to treat): (1) udder diseases (e.g., clinical mastitis); (2) reproductive failures (e.g., retained placenta and endometritis); (3) lameness; (4) digestive diseases (e.g., calf diarrhea); (5) respiratory diseases (e.g., calf pneumonia); (6) metabolic diseases (e.g., ketosis); and (7) other surgical and medical preparations. The final indication group comprised materials and treatments that could not be readily classified into other categories, including diagnostic tools and miscellaneous medical preparations. The categorization was based directly on the indications recorded in the farm treatment logs, where each administered product was linked to a specific diagnosis or health problem. When a drug was used for more than one condition, it was assigned to the indication explicitly stated in the treatment record for that particular administration.

In addition, antibiotic costs were categorized into four groups based on the indications recorded in the farm treatment logs for each specific treatment event: udder diseases, lameness, respiratory and digestive disorders, and reproductive failures (1, 2, 36, 39). Antibiotic costs were calculated on a per-cow basis. Expenditures were further classified by active substance, both within each indication category and in total. Combination antibiotics were subdivided into five groups according to their constituent active substances.

## 3 Results

### 3.1 Farm characteristics

A total of 20 commercial dairy herds participated in the study, including three from Czechia, eight from Hungary, three from Serbia, three from Slovakia, and three from Slovenia. The main production data of the farms for 2019 are shown in Table 1. A total of 20 herds were examined, the total number of cows was 18,653, with an average of 933 ± 544 (*n* = 20; min. 189, max. 2,038). Housing technology was characterized by freestall systems in all herds except for one in Slovakia. Cows with certain diseases (e.g., cases of clinical mastitis) were kept in separate barns on 41.2% of the examined farms. The milking systems used included Alfa Laval (35.3%), DeLaval (23.5%), Baumatic (17.6%), Fullwood (11.8%), SAC and Westfalia (each 5.9%). All herds were free of tuberculosis, brucellosis and bovine leukosis. In addition, 47.1% of the herds were free from infectious bovine rhinotracheitis (IBR), 41.2% were free from bovine viral diarrhea, and 5.9% of the farms were free from paratuberculosis, salmonellosis, and cryptosporidiosis.

**Table 1:** Yearly production data of the surveyed dairy farms by country (2019).

**Indexes**	**CZE (*n* = 3,200)**	**HUN (*n* = 7,025)**	**SRB (*n* = 4,613)**	**SVK (*n* = 3,043)**	**SVN (*n* = 774)**
Number of herds	3	8	3	3	3
Average number of cows	1,067 ± 611; min. 400; max. 1,600	878 ± 490; min. 469; max. 1,973	1,538 ± 470; min. 1,105; max. 2,038	1,014 ± 301; min. 681; max. 1,265	257 ± 90; min. 189; max. 359
Annual milk production (kg/farm)	3,692,500 ± 410,800; min. 3,230,000; max. 4,015,000	8,523,768 ± 5,465,600; min. 4,374,294; max. 21,020,980	No data	10,539,503 ± 3,155,873; min. 6,904,751; max. 12,582,639	2,571,585 ± 859,102; min. 1,910,968; max. 3,542,788
Marketed/produced milk (%)	94.2; min. 92.0, max. 95.5	98.1; min. 96.9; max. 100.0	No data	98.1; min. 97.4; max. 98.5	94.2; min. 91.3; max. 95.9
Average milk yield per lactation (kg)	No data	10,547 ± 1,298; min. 9,200; max. 12,505	No data	11,097 ± 303; min. 10,883; max. 11,311	10,938 ± 437; min. 10,548; max. 11,411
Average milk yield per year (kg)	No data	9,580 ± 974; min. 8,302; max. 10,900	No data	10,870 ± 675; min. 10,139; max. 11,470	10,506 ± 223; min. 10,250; max. 10,652
Average milk fat (%)	3.82 ± 0.09; min. 3.75; max. 3.92	3.75 ± 0.24; min. 3.50; max. 4.20	No data	3.71 ± 0.11; min. 3.59; max. 3.80	3.72 ± 0.04; min. 3,68; max. 3.76
Average milk protein (%)	3.29 ± 0.21; min. 3.10; max. 3.51	3.32 ± 0.10; min. 3.20; max. 3.50	No data	3.22 ± 0.10; min. 3.11; max. 3.30	3.31 ± 0.02; min. 3.29; max. 3.33
Average SCC	190,000 ± 55,678; min. 130,000; max. 240,000	382,875 ± 117,412; min. 280,000; max. 636,000	No data	263,433 ± 24,736; min. 235,000; max. 280,000	310,000 ± 17,321; min. 300,000; max. 330,000
Average length of lactation (days)	314 ± 16; min. 305; max. 332	375 ± 38; min. 320; max. 415	No data	344 ± 19; min. 321; max. 354	326 ± 11; min. 313; max. 334
Average length of calving interval (days)	404 ± 9; min. 398; max. 415	398 ± 59; min. 278; max. 494	No data	398 ± 15; min. 387; max. 408	395 ± 9; min. 385; max. 401
Number of culled cows per year	273 ± 150; min. 120; max. 420	299 ± 179; min. 160; max. 664	No data	344 ± 92; min. 259; max. 441	85 ± 31; min. 57; max. 119

CZE, Czechia; HUN, Hungary; SRB, Serbia; SVK, Slovakia; SVN, Slovenia.

The “n” indicates the total number of cows surveyed in each country.

### 3.2 Distribution of drug costs by product group

The distribution of drug costs by product group in 2019 is shown in Table 2. Antibiotics accounted for the largest proportion of costs in all countries, peaking in Slovenia (55.5%) and being lowest in Hungary (35.9%). Hormones represented a particularly high share in Czechia (24.7%) and Slovakia (22.2%), whereas their share was much smaller in Slovenia (8.7%). Surgical and medical preparations were most notable in Serbia (20.6%) and Slovenia (19.4%), while vaccines had a considerable share in Hungary (19.5%).

**Table 2:** Distribution of drug costs by product group and country in 2019 (€, %).

**Product group**	**CZE (*****n*** = **3,200)**			**HUN (*****n*** = **7,025)**			**SRB (*****n*** = **4,613)**			**SVK (*****n*** = **3,043)**			**SVN (*****n*** = **774)**		
	€**/year**	€**/cow/year**	**%**	€**/year**	€**/cow/year**	**%**	€**/year**	€**/cow/year**	**%**	€**/year**	€**/cow/year**	**%**	€**/year**	€**/cow/year**	**%**
Antibiotics	44,039.9	13.8	47.5	183,762.5	26.2	35.9	106,135.4	23.0	41.8	105,307.0	34.6	43.0	42,389.3	54.9	55.5
Hormones	22,876.3	7.1	24.7	96,136.0	13.7	18.8	45,604.9	9.9	18.0	54,439.2	17.9	22.2	6,653.0	8.6	8.7
Surgical and medical preparations	4,123.2	1.3	4.5	56,150.5	8.0	10.9	52,277.0	11.3	20.6	34,541.1	11.4	14.1	14,781.6	19.1	19.4
Vaccines	13,204.8	4.1	14.2	99,801.0	14.2	19.5	2,690.2	0.6	1.1	32,328.6	10.6	13.2	6,289.2	8.1	8.2
Vitamins	8,450.6	2.6	9.1	37,537.9	5.3	7.3	46,533.0	10.1	18.3	6,910.0	2.3	2.8	3,804.9	4.9	5.0
Antiparasitics	0.0	0.0	0.0	39,166.4	5.6	7.6	438.0	0.1	0.2	11,575.3	3.8	4.7	2,476.3	3.2	3.2
Total	92,694.9	29.0	100.0	512,554.2	73.0	100.0	253,678.6	55.0	100.0	245,101.3	80.5	100.0	76,394.2	99.0	100.0

CZE, Czechia; HUN, Hungary; SRB, Serbia; SVK, Slovakia; SVN, Slovenia.

The “n” indicates the total number of cows surveyed in each country.

**Figure 1:**
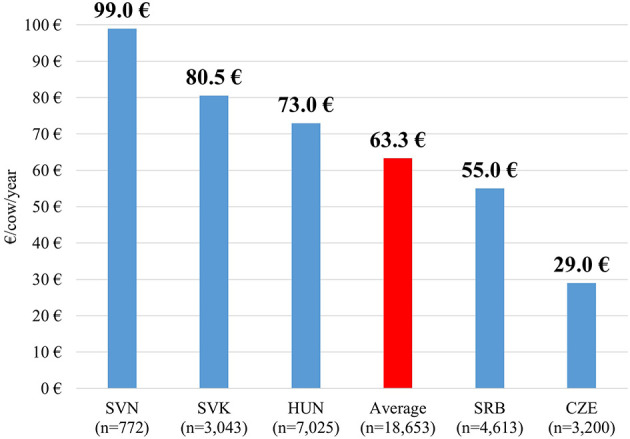
Yearly drug costs per cow by country in 2019 (€).

In 2019, the average annual veterinary drug cost per cow in the surveyed Central European herds was €63.3 (min: €14.1, max: €118.8). Among the five countries, the highest value was observed in Slovenia, followed by Slovakia and Hungary. Serbia showed a lower average, while Czechia had by far the lowest cost per cow (Figure 1).

**Figure 2:**
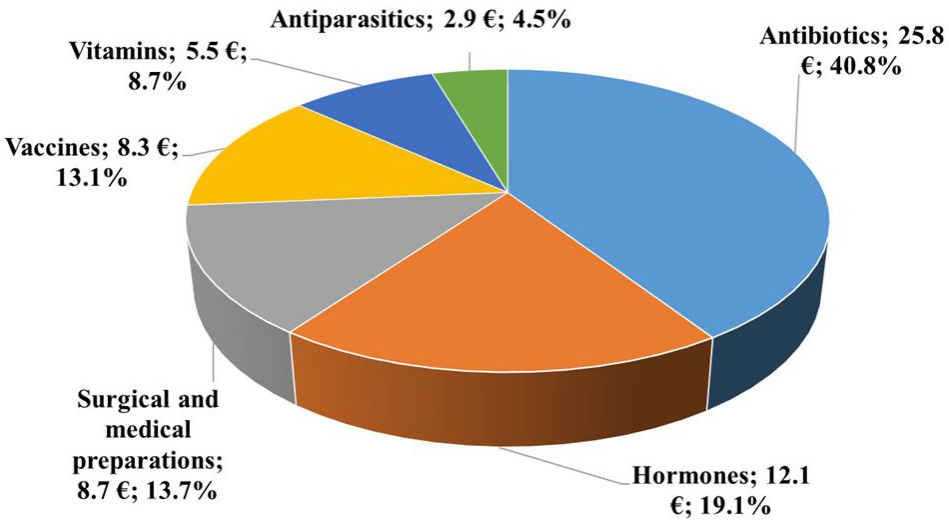
Distribution of average yearly drug costs per cow by product group in 2019 (€, %, *n* = 18,653).

Across all countries, antibiotics accounted for 40.8% of the total drug cost, which was €25.8 per cow (Figure 2). In addition to antibiotics, hormones represented about one-fifth of the total expenditures. Surgical and medical preparations and vaccines also constituted considerable shares, while vitamins and antiparasitics each accounted for less than 10% of the total costs.

### 3.3 Distribution of drug costs by indication

The distribution of veterinary drug costs by indication in 2019 is presented in Table 3. Udder diseases represented the highest proportion of costs in all countries, ranging from approximately 30% in Hungary, Serbia, and Slovakia to nearly half (48.2%) in Slovenia. Reproductive disorders were the second largest category, particularly important in Czechia (31.1%) and Slovakia (25.1%), while accounting for less than 12% in Slovenia. Lameness showed notable contributions in Serbia (13.9%) and Slovakia (16.2%), whereas respiratory diseases were most pronounced in Czechia (14.2%). Digestive diseases accounted for about 12% in Hungary but were negligible elsewhere. Metabolic diseases stood out in Serbia, comprising almost one-fifth (19.5%) of total costs, compared with ≤ 8% in other countries.

**Table 3:** Distribution of drug costs by indication and country in 2019 (€, %).

**Indication**	**CZE (*****n*** = **3,200)**			**HUN (*****n*** = **7,025)**			**SRB (*****n*** = **4,613)**			**SVK (*****n*** = **3,043)**			**SVN (*****n*** = **774)**		
	€**/year**	€**/cow/year**	**%**	€**/year**	€**/cow/year**	**%**	€**/year**	€**/cow/year**	**%**	€**/year**	€**/cow/year**	**%**	€**/year**	€**/cow/year**	**%**
Udder diseases	35,161.3	11.0	37.9	157,977.4	22.5	30.8	74,835.9	16.2	29.5	75,383.2	24.8	30.8	36,780.1	47.6	48.2
Reproductive failures	28,847.0	9.0	31.1	120,750.0	17.2	23.6	50,950.8	11.0	20.1	61,544.6	20.2	25.1	8,632.0	11.2	11.3
Lameness	8,708.6	2.7	9.4	42,562.4	6.1	8.3	35,420.5	7.7	13.9	39,719.8	13.1	16.2	7,553.2	9.8	9.9
Respiratory diseases	13,111.7	4.1	14.2	57,281.2	8.2	11.2	13,523.8	2.9	5.3	29,451.7	9.7	12.0	4,436.7	5.7	5.8
Digestive diseases	2,318.1	0.7	2.5	66,015.2	9.4	12.9	6,633.7	1.4	2.6	12,849.2	4.2	5.3	5,972.8	7.7	7.8
Metabolic diseases	3,064.2	1.0	3.3	21,733.8	3.1	4.2	49,396.0	10.7	19.5	10,847.8	3.6	4.4	6,062.2	7.9	7.9
Antiparasitics	0.0	0.0	0.0	39,166.4	5.6	7.6	438.0	0.1	0.2	11,575.3	3.8	4.7	2,476.3	3.2	3.2
Other surgical and medical preparations	1,483.9	0.5	1.6	7,067.7	1.0	1.4	22,479.7	4.9	8.9	3,729.6	1.2	1.5	4,480.9	5.8	5.9
Total	92,694.8	29.0	100.0	512,554.2	73.0	100.0	253,678.5	55.0	100.0	245,101.2	80.5	100.0	76,394.2	99.0	100.0

CZE, Czechia; HUN, Hungary; SRB, Serbia; SVK, Slovakia; SVN, Slovenia.

The “n” indicates the total number of cows surveyed in each country.

**Figure 3:**
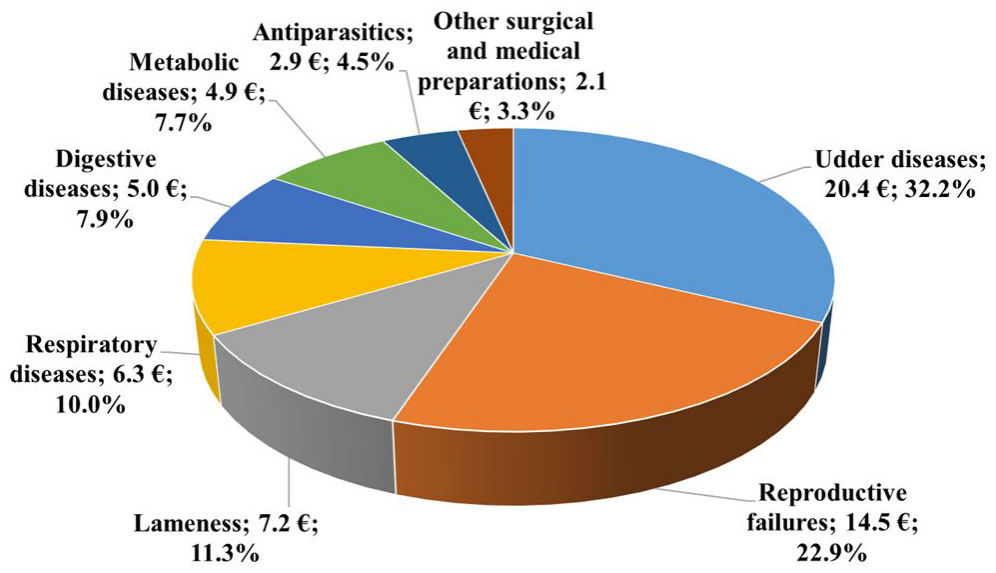
Distribution of average yearly drug costs per cow by indication in 2019 (€, %, *n* =18,653).

The average annual drug cost per cow by indication is illustrated in Figure 3. Drugs used for the treatment of udder diseases (32.2%) represented the largest share of total costs, which amounted to €20.4 per cow. Drugs used to treat reproductive failures ranked second, accounting for nearly one-fifth of the total costs. Preparations for the treatment of lameness and respiratory diseases, each with a share of about 10%, ranked third and fourth, respectively. The proportions of drugs used to treat digestive and metabolic diseases were slightly below 10%. The remaining indications accounted for nearly 8% of the total costs.

### 3.4 Distribution of antibiotic costs by indication

The distribution of antibiotic costs by indication in the surveyed dairy farms is shown in Table 4. Udder diseases represented the highest share in all countries, accounting for 60%−67% in Hungary, Serbia, Slovenia, and Czechia, and approximately 50% in Slovakia. Lameness was the second major category, with particularly high proportions in Slovakia (34.8%) and Hungary (21.7%). Respiratory and digestive diseases contributed most notably in Czechia (14.8%) and Serbia (12.4%) but remained below 10% in Slovenia. Reproductive failures generally accounted for only a minor share, ranging from 1 to 7% across the countries.

**Table 4:** Distribution of antibiotic costs by indication and country in 2019 (€, %).

**Indication**	**CZE (*****n*** = **3,200)**			**HUN (*****n*** = **7,025)**			**SRB (*****n*** = **4,613)**			**SVK (*****n*** = **3,043)**			**SVN (*****n*** = **774)**		
	€**/year**	€**/cow/year**	**%**	€**/year**	€**/cow/year**	**%**	€**/year**	€**/cow/year**	**%**	€**/year**	€**/cow/year**	**%**	€**/year**	€**/cow/year**	**%**
Udder diseases	28,212.7	8.8	64.1	109,444.6	15.6	59.6	70,449.7	15.3	66.4	52,368.7	17.2	49.7	28,608.3	37.1	67.5
Lameness	8,708.6	2.7	19.8	39,874.0	5.7	21.7	20,601.2	4.5	19.4	36,667.3	12.0	34.8	7,286.5	9.4	17.2
Respiratory and digestive diseases	6,534.3	2.0	14.8	21,024.9	3.0	11.4	13,192.6	2.9	12.4	10,141.0	3.3	9.7	4,590.0	5.9	10.8
Reproductive failures	584.2	0.2	1.3	13,419.0	1.9	7.3	1,891.9	0.4	1.8	6,130.1	2.0	5.8	1,904.6	2.5	4.5
Total	44,039.9	13.8	100.0	183,762.5	26.2	100.0	106,135.4	23.0	100.0	105,307.0	34.6	100.0	42,389.3	54.9	100.0

CZE, Czechia; HUN, Hungary; SRB, Serbia; SVK, Slovakia; SVN, Slovenia.

The “n” indicates the total number of cows surveyed in each country.

**Figure 4:**
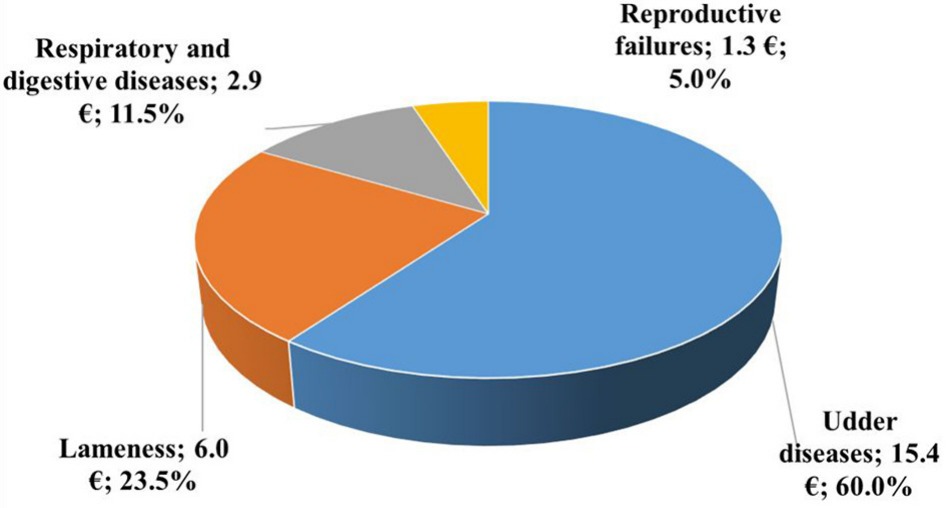
Distribution of average yearly antibiotic costs per cow by indication in 2019 (%, €, *n* = 18,653).

The distribution of the average annual costs of antibiotics per cow is illustrated in Figure 4. Preparations for the treatment of udder diseases accounted for 60% of the annual expenditure on antibiotics in the herds, corresponding to €15.4 per cow. Antibiotics used to treat lameness represented almost one-fifth of the total antibiotic costs. By contrast, the cost shares of antibiotics for the treatment of respiratory and digestive diseases, and reproductive failures were lower.

**Figure 5:**
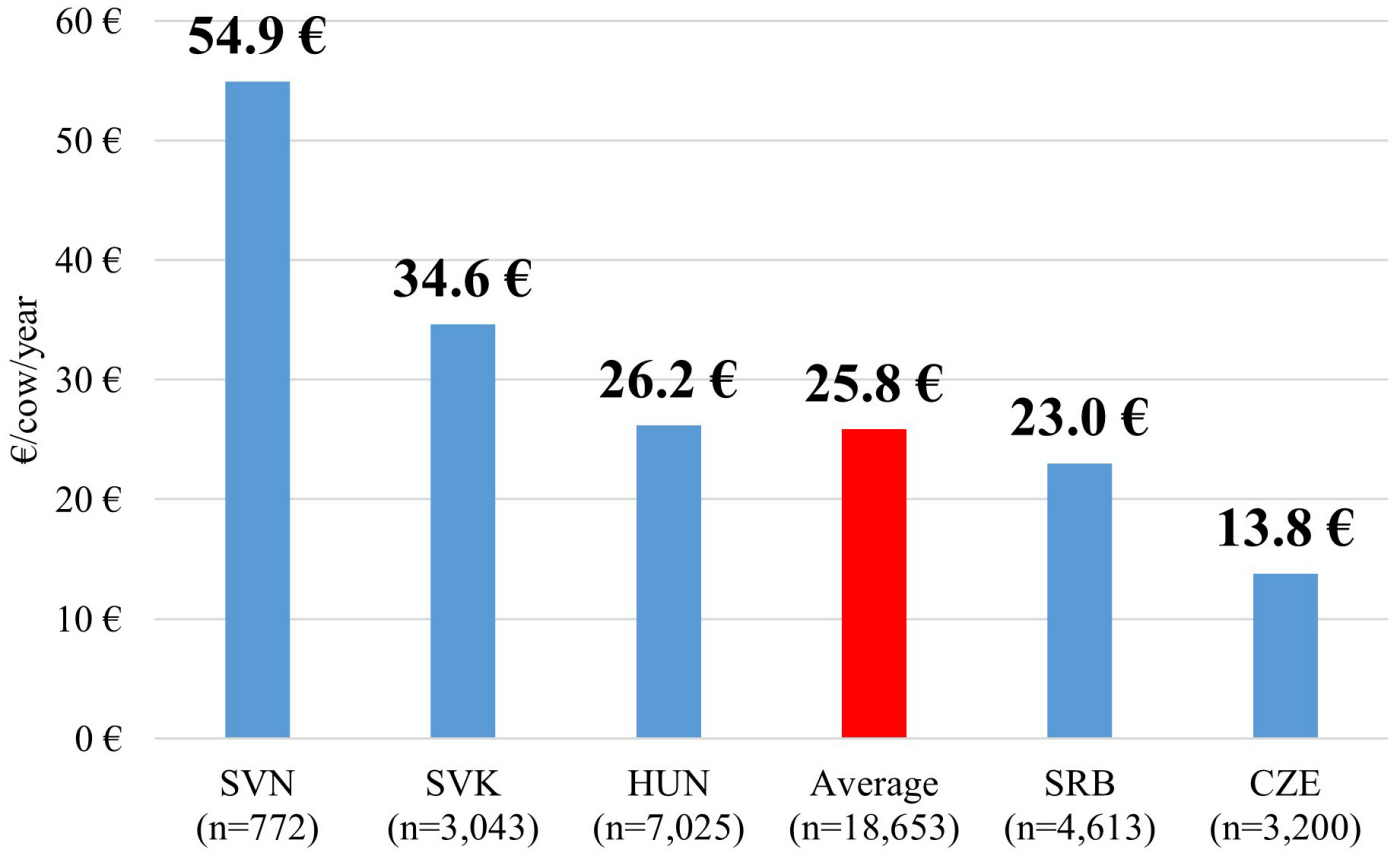
Yearly antibiotic costs per cow by country in 2019 (€).

The average annual antibiotic cost per cow across the five Central European countries was €25.8 (min: €7.2, max: €62.9). Slovenia recorded by far the highest value, followed by Slovakia and Hungary, all above the regional average. Serbia was slightly below the average, while Czechia had the lowest cost (Figure 5).

### 3.5 Antibiotic costs by class of active substance

The distribution of the average annual costs of antibiotics per cow by antimicrobial drug class, as well as the percentage distribution of antibiotic costs within each class, is presented in Table 5. On average, across the five countries, cephalosporins were predominantly used, accounting for more than 40% of expenditures, mainly containing ceftiofur and cefquinome. The drug cost per cow exceeded €10 only for cephalosporins. Antibiotic combinations (mainly penicillin-aminoglycoside combinations and amoxicillin-clavulanic acid) ranked second, accounting for about one-fifth of the total costs. Penicillins (mainly cloxacillin and benzylpenicillin-procaine) and fluoroquinolones (mainly marbofloxacin and enrofloxacin) ranked third and fourth, each accounting for nearly one-tenth of the total costs. Tetracyclines (mainly oxytetracycline and chlortetracycline) and macrolides (mainly tilmicosin and tulathromycin) contributed almost equally to the total antibiotic costs.

**Table 5:** Distribution of average yearly antibiotic costs by antimicrobial drug class in 2019 (€, %; *n* = 18,653).

**Antimicrobial drug classification**	**Costs (€)**	**Share (%)**	**Antibiotic costs per cow (€)**
Cephalosporins	210,423.0	43.7	11.3
Combinations^†^	102,333.3	21.3	5.5
Penicillins	46,316.6	9.6	2.5
Fluoroquinolones	42,890.5	8.9	2.3
Tetracyclines	25,057.1	5.2	1.3
Macrolides	21,778.7	4.5	1.2
Others^‡^	32,834.9	6.8	1.8
Total	481,634.1	100.0	25.9

^†^Combinations: 1. Penicillin-aminoglycoside combinations (e.g., benzylpenicillin-procaine, dihydrostreptomycin, neomycin). 2. Amoxicillin-clavulanic acid. 3. Lincosamide-aminoglycoside combinations (lincomycin, neomycin, spectinomycin). 4. Penicillin-polypeptide antibiotic combinations (amoxicillin, ampicillin, colistin). 5. Combination of other antibiotics (e.g., bacitracin, chlortetracycline, oxytetracycline).

^‡^Others: aminoglycosides, phenicols (mainly florfenicol), lincosamides (mainly lincomycin), polypeptide antibiotics, potentiated sulfonamides and other antibiotics (rifaximin).

Antibiotic costs were also analyzed by disease category, grouped according to antimicrobial drug classification. For the treatment of udder diseases (Table 6), almost equal proportions of antibiotic combinations (mainly penicillin-aminoglycoside combinations and amoxicillin-clavulanic acid) and cephalosporins (mainly cefquinome, cefoperazone, and cefapirin) were used. The cost shares of penicillins (mainly cloxacillin and benzylpenicillin-procaine) and fluoroquinolones (mainly marbofloxacin and enrofloxacin) were also considerable. Within the “Others” category, tetracyclines (oxytetracycline), lincosamides (lincomycin), and macrolides (tylosin) accounted for higher proportions than potentiated sulfonamides and aminoglycosides, although none exceeded 2% within this indication.

**Table 6:** Distribution of average yearly antibiotic costs for the treatment of udder diseases by antimicrobial drug class in 2019 (€, %; *n* = 18,653).

**Antimicrobial drug classification**	**Costs (€)**	**Share (%)**	**Antibiotic costs per cow (€)**
Combinations^†^	99,431.6	34.4	5.3
Cephalosporins	95,430.7	33.0	5.1
Penicillins	44,375.4	15.4	2.4
Fluoroquinolones	37,314.9	12.9	2.0
Others^‡^	12,531.4	4.3	0.7
TOTAL	289,083.9	100.0	15.5

^†^Combinations: 1. Penicillin-aminoglycoside combinations (e.g., benzylpenicillin-procaine, dihydrostreptomycin, neomycin). 2. Amoxicillin-clavulanic acid. 3. Lincosamide-aminoglycoside combinations (lincomycin, neomycin, spectinomycin). 4. Combination of other antibiotics (e.g., bacitracin, chlortetracycline, oxytetracycline).

^‡^Others: tetracyclines, lincosamides, sulfonamides, macrolides, aminoglycosides.

Two groups of antibiotics were used to treat lameness: cephalosporins and tetracyclines. Within the cephalosporins, ceftiofur and cefquinome predominated, while among tetracyclines, oxytetracycline was more frequently used than chlortetracycline. Cephalosporins, tetracyclines, other antibiotics (rifaximin) and penicillins were used to treat reproductive failures. Most treatments involved cefapirin, representing 85.4% of the cephalosporin class. Chlortetracycline accounted for 15.0% within this indication. The combined proportion of oxytetracycline, ceftiofur and amoxicillin was 21.2% (Table 7).

**Table 7:** Distribution of average yearly antibiotic costs for the treatment of lameness and reproductive failures by antimicrobial drug class in 2019 (€, %; *n* = 18,653).

**Indication**	**Antimicrobial drug classification**	**Costs (€)**	**Share (%)**	**Antibiotic costs per cow (€)**
Lameness	Cephalosporins	99,667.9	88.1	5.3
	Tetracyclines	13,469.6	11.9	0.7
	Subtotal	113,137.5	100.0	6.1
Reproductive failures	Cephalosporins	14,216.4	59.4	0.8
	Tetracyclines	6,152.1	25.7	0.3
	Other antibiotics	3,124.1	13.1	0.2
	Penicillins	437.3	1.8	0.0
	Subtotal	23,929.9	100.0	1.3

Macrolides (mainly tilmicosin, tulathromycin, and tildipirosin) and phenicols (florfenicol) were used in almost equal proportions for treating respiratory and digestive diseases, together accounting for more than 60% of the total costs (Table 8). Fluoroquinolones (mainly enrofloxacin) and potentiated sulfonamides (mainly sulfadoxine trimethoprim) were also widely used. Within antibiotic combinations, penicillin-polypeptide combinations represented the highest share. In the “Others” category, lincosamides (lincomycin), penicillins (amoxicillin) and cephalosporins (ceftiofur) were the most common, while polypeptide antibiotics (colistin) and tetracyclines (mainly chlortetracycline) accounted for the smallest shares.

**Table 8:** Distribution of average yearly antibiotic costs for the treatment of respiratory and digestive diseases by antimicrobial drug class in 2019 (€, %; *n* = 18,653).

**Antimicrobial drug classification**	**Costs (€)**	**Share (%)**	**Antibiotic costs per cow (€)**
Macrolides	18,431.3	33.2	1.0
Phenicols	17,944.6	32.3	1.0
Fluoroquinolones	5,575.6	10.1	0.3
Potentiated sulfonamides	4,918.5	8.9	0.3
Combinations^†^	2,901.8	5.2	0.2
Others^‡^	5,711.1	10.3	0.3
Total	55,482.8	100.0	3.1

^†^Combinations: 1. Penicillin-aminoglycoside combinations (e.g., benzylpenicillin-procaine, dihydrostreptomycin, neomycin). 2. Amoxicillin-clavulanic acid. 3. Lincosamide-aminoglycoside combinations (lincomycin, neomycin, spectinomycin). 4. Penicillin-polypeptide antibiotic combinations (amoxicillin, ampicillin, colistin). 5. Combination of other antibiotics (e.g., bacitracin, chlortetracycline, oxytetracycline).

^‡^Others: cephalosporins, penicillins, tetracyclines, lincosamides, aminoglycosides.

## 4 Discussion

### 4.1 Veterinary drug costs by product group

Understanding how veterinary drug costs are distributed among different product groups is essential for identifying priorities in disease prevention, management strategies, and economic efficiency in dairy herds. The categorization of drug costs by indication and product group correlates with production indices and overall farm profitability (2). Prevention-oriented strategies and diagnostics are increasingly recognized as key to reducing drug costs and antibiotic dependence in dairy farming, as evidenced in the Nordic countries where antibiotic use is low despite high milk quality and strong disease control practices (8). The International Dairy Federation (IDF) report highlights that many dairy farms globally are strengthening antimicrobial oversight, increasing use of diagnostics, vaccination, and herd health monitoring to minimize the need for antibiotic treatments (40).

In our study, antibiotics accounted for the largest share of drug costs by product group (approximately 40%). The proportion of antibiotics was the lowest on Hungarian farms, which may be related to the high share of preventive vaccination against diseases. At the European level, regulations aimed at curbing antimicrobial resistance (AMR) are increasingly shaping antibiotic use practices in livestock. The EU has introduced legislative measures, monitoring systems, and policy incentives to promote responsible antibiotic stewardship in food-producing animals (41, 42). In Hungary, monitoring of raw milk has confirmed the presence of antimicrobial resistance genes along the production chain, underlining that AMR is not only a regulatory concern but a measurable risk within dairy farming (43). Similarly, recent analyses from Slovak dairy farms have demonstrated antimicrobial resistance in *Escherichia coli* isolates, highlighting the link between antibiotic use patterns and the emergence of resistance at herd level (44). Earlier studies from Slovakia also reported the prevalence of mastitis pathogens and their antimicrobial susceptibility, emphasizing the need for evidence-based treatment approaches (27). These findings highlight that antimicrobial resistance is a major public health issue that is shaped not only by policy and regulation but also by the practical, day-to-day realities faced by Central European dairy farmers and veterinarians, directly affecting treatment decisions and herd-level health management.

A considerable share of costs (19.1%) was spent on hormones for the treatment of reproductive failures (22.9%), mainly in Czech and Slovak farms. The higher use of hormone-related expenditures in these herds may be linked to differences in reproductive management strategies compared with the other participating countries. In Czech and Slovak dairy systems, veterinarians regularly report conducting fertility examinations and the frequent use of hormonal protocols for oestrus synchronization and timed artificial insemination, which is in line with findings from studies on veterinary herd health management and reproductive hormone use in Western European dairy herds (45, 46). The average yearly drug cost per cow to treat reproductive failures was €14.5 across the Central European dairy farms. Kaneene and Hurd calculated a higher value (€19.2) (47), while Ózsvári and Kerényi reported a lower value (€12.3) (48).

The share of surgical and medical preparations was 13.7% on average, with the highest in Serbian and Slovenian farms. According to a study by Wilm et al., almost all veterinarians interviewed reported that they often or always used non-steroidal anti-inflammatory drugs (NSAID) as adjunctive therapy for mastitis (49). The high prevalence of mastitis and lameness in Slovak farms may explain the elevated use of surgical and medical preparations. Recent randomized trials have shown that combining non-steroidal anti-inflammatory drugs (e.g., ketoprofen) with therapeutic hoof trimming and hoof block application significantly improves lameness recovery in multiparous dairy cows compared to trimming alone (50).

Among the participating countries, the proportion of vaccine-related costs was lowest in Serbian herds and highest in Hungarian dairies. However, these values reflect expenditures only and do not necessarily correspond to actual vaccination practices, as vaccine usage cannot be inferred from cost data alone. Respiratory and digestive diseases remained common on these farms. Bovine respiratory disease (BRD) most often develops before or shortly after weaning in calves, when maternal antibody titres may be low in some animals, necessitating vaccination at or before weaning. Windeyer et al. found that the effectiveness of vaccination does not depend on herd size, region, season of birth or herd-level prevalence of BRD (51). Dairy herds across Europe apply a range of preventive vaccinations, but BRD remains one of the most important calf health problems even in herds that routinely vaccinate against major respiratory pathogens such as *Pasteurella multocida, Mannheimia haemolytica, Mycoplasma* spp., Bovine Respiratory Syncytial Virus and Bovine Herpesvirus Type 1 (52, 53). Incompletely matched vaccine spectra, suboptimal timing in relation to maternal antibody decline, and management and environmental risk factors may all limit the preventive impact of vaccination and sustain the need for antibiotic treatments (52–55).

Vitamins were administered in connection with the prevention and treatment of metabolic diseases. The comparatively higher vitamin-related expenditures observed in Serbian herds may reflect differences in recorded metabolic conditions and/or a different preventive or therapeutic supplementation approach at herd level (34). The cost per cow for metabolic diseases was €4.9, which is similar to the value reported by Kaneene and Hurd (€4.7) (47).

In our study, antiparasitic treatments represented the lowest share (4.5%) of drug costs among the product groups. On a European scale, parasitic infections in ruminants are estimated to cost the industry around €1.8 billion annually, of which approximately 20% stems from treatment costs and the remainder from production losses (56). However, these sector-level estimates are not directly comparable to our farm-level data, which reflect only veterinary drug expenditures, without considering other treatment-related costs.

The average annual drug cost per cow was €63.3. Comparison with other studies is complicated by differences in data collection methods and time periods (30). Steeneveld et al. (57) also emphasized that veterinary drug expenditures should be interpreted within the broader context of animal health costs, which include both direct treatment expenses and indirect production losses. Previous Hungarian surveys reported a wide range of values (€41.8–95.3) (1, 2, 39). Yilmaz et al. calculated €63.8 for four Turkish farms (58). This estimate included veterinary labor costs in addition to the drug costs but was almost equal to the value we reported. In a broader international comparison, on-farm health costs have been shown to vary substantially between countries and production systems, depending on how drug, labor, and disease-related losses are accounted for (59). Slovenian farms had the highest average yearly drug cost per cow, largely due to the high cost of antibiotics and udder disease treatments. Recent estimates suggest that the total annual economic burden of cattle diseases in the EU can exceed €185 per cow when production losses are included (60).

Although our study did not assess economic indicators such as milk revenue or total production costs, previous studies (2, 39) suggest that total veterinary drug costs typically account for approximately 1.5%−2.5% of total production costs in large commercial Hungarian dairy herds, with antibiotic expenditures representing roughly 0.4%−0.6% of this value. These proportions indicate that, while antibiotics form the largest share within the veterinary drug budget, they represent only a small fraction of overall production costs.

### 4.2 Veterinary drug costs by indication

Our results show that the largest share of drug costs by indication was allocated to the treatment of udder diseases, ranging from 30 to 50% across countries. The highest proportions were found in Czech and Slovenian dairies, while Serbian farms reported the lowest values. These differences may be explained by variations in housing technologies, milking systems, and the availability of paddocks (61). In general, variation in the bacterial species causing mastitis can influence case severity and the choice of therapeutic protocols, which in turn may affect treatment costs (62). However, bacteriological testing was not available in our dataset, and therefore country-specific pathogen differences cannot be evaluated in this study. The average cost per cow for udder health was €20.4, whereas higher values were reported by Kaneene and Hurd (47) (€27.9) and van Soest et al. (61) (€34). These differences may reflect differences in herd size, management systems, disease definitions, or cost-calculation methods (63).

Reproductive disorders represented the second-largest share of drug costs across countries (€14.5/cow), with comparatively higher proportions in Czech and Slovak herds. In our dataset, expenditures were mainly associated with therapies for postpartum uterine disorders and cycle management. Recent herd-level estimates indicate that interrelated reproductive problems can impose substantial annual costs, on the order of €100 per cow when multiple disorders co-occur, while single-disorder costs vary widely (e.g., €30/cow/year for acute metritis), depending on incidence and case management (64). These published values include total economic losses (e.g., reduced milk yield, impaired fertility and increased culling), whereas our estimates reflect drug costs only, which explains why the literature values appear higher and are not directly comparable (3).

The cost share of preparations used to treat lameness was also substantial (11.3%), with the highest values recorded in Slovak and Serbian herds and the lowest in Hungarian dairies. The higher prevalence of lameness in some herds may be linked to less frequent hoof care, which was only provided when deemed necessary by the farm manager, in contrast to farms where all cows received hoof trimming once or twice annually as part of a routine maintenance programme (65). The average cost per cow for lameness was €7.2, compared with a lower value of €5.3 reported by Kaneene and Hurd (47). For respiratory and digestive diseases, the average costs per cow were €6.3 and €5.0, respectively. Kaneene and Hurd (47) reported lower costs for respiratory diseases (€3.1) and higher costs for digestive disorders (€8.7). These differences likely reflect variation in management systems and treatment protocols, disease definitions, age groups included (e.g., preweaned vs. weaned calves), recording practices, and whether published values represent drug costs only or total treatment-related expenditures (9, 15, 63).

In our study, the average drug cost per cow for metabolic conditions was €4.9. Transition-related diseases (e.g., milk fever, ketosis) may entail both overt and hidden losses, and monitoring and prevention during the ±3-week window around calving are often pivotal for reducing treatment needs and associated costs. Emerging tools (e.g., mid-infrared-based screening) may support herd-level risk detection where routine blood profiling is impractical (66). In addition, precision dairy monitoring systems based on automated sensors can complement routine clinical observation by continuously tracking behavioral and physiological changes at cow level, enabling earlier recognition of developing health problems (67).

### 4.3 Antibiotic costs by indication

In our study, the largest share of antibiotic costs was consistently linked to the treatment of udder diseases (around 60%), followed by lameness, respiratory and digestive disorders, and reproductive failures across the five countries. This pattern is in line with international observations (68–72), although many of them have also examined the prevalence of other problems (e.g., septicaemia, dermatitis, ketosis, reticuloperitonitis), making it difficult to compare distributions and allowing only frequency-based comparisons. For instance, Ferroni et al. (68) reported that in Central Italian herds, 41% of treatments targeted udder health, while respiratory, reproductive, digestive, and locomotion disorders accounted for smaller shares (68). Similarly, Merle et al. found that German herds ranked udder, respiratory, and reproductive disorders among the top three, with digestive diseases also contributing substantially (69). Studies in Switzerland and Pennsylvania confirmed the predominance of udder health problems, followed by lameness, reproductive, respiratory, and digestive disorders (70, 71). In Quebec, mastitis and retained placenta were most frequent among cows, whereas pneumonia and diarrhea were leading problems in calves (72).

These findings suggest that antibiotic use in dairy herds is primarily driven by udder health management, with lameness and reproductive problems representing further important cost factors (30). Lameness in particular causes severe economic losses through premature culling, reduced milk yield, weight loss, and high treatment costs, while also impairing fertility (73). Similarly, clinical endometritis compromises reproductive performance and profitability (74). Among calves, the Bovine Respiratory Disease Complex and diarrhea remain the leading health issues, with substantial impacts on mortality, welfare, and treatment needs (75, 76).

In our study, lameness accounted for a notable share of antibiotic costs; however, recent evidence indicates that many lameness cases can be managed effectively without routine antibiotic use. A 34-month randomized controlled trial showed that combining a non-steroidal anti-inflammatory drug (NSAID; e.g., ketoprofen) with therapeutic hoof trimming and hoof block application significantly improved recovery compared with trimming alone, supporting analgesia-centered, non-antibiotic protocols for claw horn lesions (50). Sectoral guidance likewise emphasizes prompt hoof care, biosecurity, and environmental measures for infectious claw disease, with farmers reporting welfare and economic benefits from alternative, non-antibiotic treatments (40). Digital health or Precision Livestock Farming tools further enable earlier, targeted interventions that can reduce antimicrobial use while maintaining productivity (77).

The average antibiotic cost per cow in our survey was €25.8 which lies between €19.6 and 42.8 reported in earlier Hungarian studies (1, 39). On Dutch farms, costs were considerably higher (€70–84 per cow between 2005 and 2012), partly because veterinary labor costs were included (30). Among the countries examined in our study, Slovenian farms showed the highest antibiotic costs per cow, reflecting the frequent use of combined antibiotics and cephalosporins for udder disease therapy. Hungarian farms, by contrast, showed values close to the overall average, both in the cost shares by indication and in the per-cow expenditure.

Country-level patterns also demonstrated differences in antimicrobial substance use. A Czech study reported that some herds had implemented on-farm culture-based decision tools and selective dry-cow therapy, but these findings do not reflect nationwide antimicrobial protocols and should therefore not be interpreted as the cause of country-level differences (35). In Hungary, a recent study reported the preventive use of probiotics, which may indirectly lower antibiotic demand (34). A Slovak study documented the prevalence and antimicrobial susceptibility of mastitis pathogens, supporting evidence-based therapeutic choices (27). In Slovenia, despite research on genetic markers related to somatic cell counts (SCC) that supports long-term breeding strategies for udder health (78), our findings showed the highest antibiotic use among the five countries, largely driven by mastitis treatment. By contrast, although sales data from Serbia between 2017 and 2020 indicated increasing antimicrobial consumption in food-producing animals, our results showed relatively lower antibiotic costs on Serbian dairy farms, suggesting possible differences between national sales trends and herd-level expenditure patterns (79).

The observed between-country differences in veterinary drug costs and antibiotic use are likely multifactorial and should be interpreted in the context of broader structural and policy-related factors. When placing our findings within the wider One Health context, integrated European analyses have demonstrated positive associations between antimicrobial consumption and resistance in bacteria from both humans and food-producing animals, underscoring the need to reserve antibiotics for clearly indicated cases (80). International public-health bodies likewise emphasize prevention-first approaches and coordinated stewardship across sectors to limit AMR spread (80, 81). European countries differ in the design and implementation of their One Health AMR action plans and sector-specific stewardship measures, which can influence prescribing behavior and the choice of antimicrobial classes at herd level (40–42, 80, 81).

The organization of veterinary services and herd health programmes varies across regions, with some production systems placing greater emphasis on structured herd health management, benchmarking and regular veterinary advisory services (30, 40, 45, 59). Such differences in service provision may also influence disease prevention, diagnostic approaches and treatment strategies in dairy herds across the surveyed countries. Furthermore, differences in herd size, production intensity and farming systems may contribute to variation in disease pressure and treatment needs, as shown by studies reporting higher antimicrobial use in more intensive or high-yielding dairy systems compared with less intensive or alternative production systems (26, 31, 59, 82).

### 4.4 Antibiotic costs by active substance classification

In our study, cephalosporins accounted for the largest share of antibiotics (43.7%), confirming their widespread systemic use in line with previous studies (67, 82). The high reliance on 3rd and 4th generation cephalosporins is partly explained by their short or zero-day milk withdrawal periods (83). Besides cephalosporins, antibiotic combinations, penicillins, fluoroquinolones, tetracyclines, and macrolides were also frequently used, consistent with international findings (67, 70, 71, 84). For mastitis and dry-cow therapy, previous studies have reported that farms mainly used cephalosporins (cefquinome, cefoperazone, and cefapirin), fluoroquinolones, penicillins, and antibiotic combinations (e.g., amoxicillin-clavulanic acid, 1st, and 2nd generation cephalosporin-aminoglycoside combinations) (30, 67, 85, 86). Although intramammary β-lactams remain widely used for the treatment of mastitis, resistance levels vary considerably across regions and herds. A Canadian study has reported relatively low resistance among *Staphylococcus aureus* isolates; however, these findings are context-specific and cannot be generalized to all countries or production systems (87).

In our survey, ceftiofur, cefquinome, and tetracyclines were most frequently used to treat lameness, similar to practices in Wisconsin and Pennsylvania (70, 88). Cefquinome has shown high cure rates for digital dermatitis (89, 90), while oxytetracycline, often applied as a spray or in combination with sodium hypochlorite (86.7% efficacy) has also proved effective (73, 91).

For respiratory diseases, macrolides (tilmicosin, tulathromycin, and tildipirosin), phenicols (florfenicol), fluoroquinolones (enrofloxacin), potentiated sulfonamides (e.g., sulfadoxine trimethoprim), and antibiotic combinations (e.g., penicillin-polypeptide combinations) were the predominant choices in the surveyed herds, consistent with previous studies (75, 92–94). According to other studies, tulathromycin improved both survival to first calving and growth in treated calves (75), while florfenicol remained effective as *Pasteurella multocida* shows little resistance (95).

For digestive diseases, enrofloxacin and potentiated sulfonamides were the most commonly used antibiotics across the surveyed Central European farms, consistent with findings from Argentina and California (92, 93). The efficacy of fluoroquinolones has been clearly established for calf diarrhea, with oral and parenteral formulations of enrofloxacin and oral marbofloxacin approved in Europe (96, 97). These agents have a high bioavailability and are particularly effective against Gram-negative bacteria due to their broad-spectrum bactericidal activity. Potentiated sulfonamides have shown effectiveness when administered before symptoms appear and are also considered effective against salmonellosis (98). However, the use of antibiotics in a metaphylactic or prophylactic manner carries an increased risk of antimicrobial resistance development, particularly when treatments are applied to groups of animals without confirmed infection. Therefore, such approaches should be used cautiously and only when justified by clinical or epidemiological indications (80, 81).

For reproductive failures, cefapirin, ceftiofur, tetracyclines, and rifaximin were the most commonly used antibiotics in the surveyed farms. A possible explanation for the high use of cefapirin is its association with the significant increase in relative pregnancy rate in cows with subclinical endometritis treated with this antibiotic and cloprostenol (99). Other studies have also reported a significant use of tetracyclines (88), ceftiofur (70, 100) and rifaximin for treating reproductive disorders. Boudelal et al. (74) found that the combination of cefacetrile and rifaximin resulted in higher cure rates among cows with clinical endometritis and a shorter time to resumption of ovarian activity after calving (74). Additionally, intrauterine infusions containing ceftiofur have been shown to reduce the incidence of clinical endometritis (101).

Future regulation may increasingly adapt to AMR prevalence and societal expectations, making veterinarian involvement crucial. In the Netherlands, for instance, the annual herd health management programme facilitates communication between farmers and veterinarians and positively impacts both cow health and medicine use (30). Growing awareness of the negative consequences of excessive drug use has already contributed to a shift from cephalosporins and fluoroquinolones toward penicillins and narrower-spectrum antibiotics in several countries (30). Our findings confirm that intensive dairy farms are associated with high antibiotic consumption, highlighting the need to strengthen surveillance systems and promote stewardship to curb AMR. Since 2024, all EU/EEA member states have been required to report both sales and on-farm use of veterinary antimicrobials. These mechanisms underpin efforts to restrict the use of critically important antimicrobials (such as 3rd and 4th generation cephalosporins and fluoroquinolones) and to promote narrower-spectrum alternatives (23).

### 4.5 Limitations

This study uses pre-COVID data and therefore does not capture potential shifts in veterinary regulations, disease management practices, or market conditions that may have occurred during or after the pandemic. The dataset includes more than 18,000 cows from 20 large commercial farms across five Central European countries (Czechia, Hungary, Slovakia, Slovenia, and Serbia), which enhances regional representativeness but may still limit generalizability, as specific farm-level practices can bias cost estimates. Reported drug costs were converted into euros using contemporaneous prices and exchange rates (Hungarian Forint, Czech Koruna, and Serbian Dinar); subsequent inflation, supply-chain disruptions, and product availability may have affected absolute values. Moreover, because indication-based categorization relied on farm treatment logs, differences in diagnostic practices between farms and countries may have influenced the results. Additionally, data on antiparasitic drugs were not available for Czech farms, which restricts comparability for this product group. Future research including more farms and multi-year, post-COVID data would provide stronger evidence on how antibiotic-use patterns and veterinary drug expenditures have evolved.

## 5 Conclusions

In Central European dairy farms, most veterinary drug spending was on antibiotics and hormones, mainly to treat udder diseases, reproductive failures, lameness, and respiratory and digestive disorders, in line with earlier findings. We found notable cross-country differences in drug use, shaped by national practices and drug availability. Among antibiotics, cephalosporins (especially ceftiofur and cefquinome) and combinations such as penicillin-aminoglycoside and amoxicillin-clavulanic acid accounted for the largest shares. The heavy reliance on 3rd- and 4th-generation cephalosporins, classified as Highest Priority Critically Important Antimicrobials (HPCIAs), together with substantial intramammary use, highlights key targets for reduction and improved antimicrobial stewardship. Sustained veterinary oversight, regular herd health monitoring, and prevention-focused management, supported by careful selection of antimicrobial classes, are essential to reduce disease incidence and antimicrobial use. For policymakers, the observed cross-country differences underline the need for harmonized monitoring and targeted national strategies that lower dependence on HPCIAs while maintaining economically viable prevention practices. Because our dataset reflects the situation in 2019, before major regulatory and market changes of the post-COVID period, these results provide a valuable baseline for cross-country comparisons and for assessing post-2020 changes in veterinary drug expenditures, shifts away from HPCIAs, and progress toward more preventive, health-oriented herd management in the region.

## Data Availability

The original contributions presented in the study are included in the article/supplementary material, further inquiries can be directed to the corresponding author.
