# Correction: Economic costs of veterinary drug and antibiotic use in commercial dairy cattle herds in Central European countries

**DOI:** 10.3389/fvets.2026.1870032

**Published:** 2026-05-19

**Authors:** László Ózsvári, Attila Dobos, Marietta Máté

**Affiliations:** 1 Department of Veterinary Forensics and Economics, University of Veterinary Medicine Budapest, Budapest, Hungary; 2 National Laboratory of Infectious Animal Diseases, Antimicrobial Resistance, Veterinary Public Health and Food Chain Safety, University of Veterinary Medicine Budapest, Budapest, Hungary; 3 Private Practitioner, Vác, Hungary

**Keywords:** antibiotics, antimicrobials, Central-Europe, classification of antibiotics, dairy cattle, indication of antibiotics, veterinary drug cost

Author Attila Dobos was omitted as an author in the published article. The correct author list reads:

László Ózsvári^1,2^^*^, Attila Dobos^3^ and Marietta Máté^1, 2^

^1^Department of Veterinary Forensics and Economics, University of Veterinary Medicine Budapest, Budapest, Hungary

^2^National Laboratory of Infectious Animal Diseases, Antimicrobial Resistance, Veterinary Public Health and Food Chain Safety, University of Veterinary Medicine Budapest, Budapest, Hungary

^3^Private Practitioner, Vác, Hungary

The **Author contributions** section has been corrected to read:

LÓ: Conceptualization, Methodology, Validation, Investigation, Resources, Writing – original draft, Writing – review & editing, Supervision, Project administration, Funding acquisition. AD: Conceptualization, Validation, Investigation, Writing – review & editing. MM: Conceptualization, Methodology, Validation, Formal analysis, Data curation, Writing – original draft, Writing – review & editing, Visualization.

As Attila Dobos has now been added as an author, the following sentence has been removed from the **Acknowledgments** section: “The Authors would like to thank Attila Dobos for his contribution to the data collection.”

The original version of this article has been updated.

